# Novel Insights on Clinical Outcomes Using Integrated Shotgun Metagenomic Profiling of the Gut Microbiome, Resistome, and Host Immune-Inflammatory Response in Hospitalized Patients with Decompensated Cirrhosis

**DOI:** 10.3390/pathogens15030241

**Published:** 2026-02-24

**Authors:** Cyriac Abby Philips, Tharun Tom Oommen, Arif Hussain Theruvath, Aryalakshmi Sreemohan, Ambily Baby, Ansu Abu Alex, Sunitha Thomas, Sunitha Mary John, Rizwan Ahamed, Ajit Tharakan, Philip Augustine

**Affiliations:** 1 Department of Clinical and Translational Hepatology, The Liver Institute, Center of Excellence in Gastrointestinal Sciences, Rajagiri Hospital, Aluva 683112, Kerala, India; 2 Clinical Research Division, The Liver Institute, Center of Excellence in Gastrointestinal Sciences, Rajagiri Hospital, Aluva 683112, Kerala, India; 3 Department of Gastroenterology and Advanced GI Endoscopy, Center of Excellence in Gastrointestinal Sciences, Rajagiri Hospital, Aluva 683112, Kerala, India; 4 Department of Advanced Flow Cytometry Immunophenotyping Facility, Rajagiri Hospital, Aluva 683112, Kerala, India; 5 Department of Pathology and Laboratory Services, Rajagiri Hospital, Aluva 683112, Kerala, India; 6 Department of Biochemistry, Rajagiri Hospital, Aluva 683112, Kerala, India

**Keywords:** decompensated cirrhosis, portal hypertension, microbiota, metagenomics, sepsis

## Abstract

**Background and Aims:** Sepsis drives mortality in cirrhosis, yet the gut antimicrobial resistance (AMR) landscape remains unmapped in high-burden settings like India. This study aimed to integrate shotgun metagenomics with deep immunophenotyping to define the gut–immune–resistome axis and correlate specific microbial and genetic signatures with clinical outcomes in decompensated cirrhosis. **Methods:** We analysed 78 hospitalized patients with cirrhosis using stool shotgun metagenomics, multiplex cytokine arrays, and flow cytometry. The microbiome and resistome (AMR genes) were mapped and correlated with disease severity, immune function (monocyte HLA-DR, neutrophil CD64), and clinical endpoints including mortality. **Results:** Disease severity was characterized by a “Gram-negative bloom” (*Klebsiella*) alongside pathogenic *Enterococcus* expansion and novel markers: *Clostridium* sp. C5-48 (severe decompensation) and *Sutterella* (ascites). A specific, dense resistome predicted adverse outcomes; the quinolone-resistance gene *QnrB4* correlated with mortality and immune paralysis, while the carbapenemase *OXA-833* gene was linked to gastrointestinal bleeding. Notably, the commensal *Ligilactobacillus salivarius* was associated with systemic inflammatory cytokines. **Conclusions:** This study reveals a “pathogenic ecosystem” in Indian decompensated cirrhosis where the resistome is intrinsically linked to host immune failure. The identification of specific prognostic markers (*QnrB4*, *OXA-833*) and inflammatory associations with *L. salivarius* challenges generic probiotic use and underscores the urgent need for precision, resistome-targeted therapies.

## 1. Introduction

Cirrhosis is no longer viewed merely as a localized fibrotic disease of the liver but as a complex, multi-systemic syndrome driven by profound immunological and hemodynamic derangements. In the modern clinical landscape, mortality in advanced liver disease is frequently precipitated by extra-hepatic organ failure and sepsis rather than liver failure alone [1]. Individuals with liver failure are at increased risk for bacterial sepsis because of immune dysregulation. Bacterial infections occur in up to 80% of these patients and are linked to a higher incidence of complications and adverse outcomes [2]. Recent data from Northern India indicates that refractory septic shock is the leading cause of mortality in hospitalized cirrhosis patients, accounting for approximately 69% of deaths [3]. The pathophysiology of this sepsis-driven mortality is rooted in the collapse of the gut–liver axis. In cirrhosis, portal hypertension and compromised intestinal barrier, increases gut permeability to bacteria and pathogen-associated molecular patterns (PAMPs) such as lipopolysaccharide (LPS). Furthermore, the development of portosystemic shunting in advanced portal hypertension creates a direct vascular bypass of the hepatic sinusoidal system, allowing gut-derived bacteria and endotoxins to circumvent the reticuloendothelial clearance function of hepatic Kupffer cells. This portosystemic shunting effectively eliminates a critical line of innate immune defense, directly promoting systemic bacteremia and endotoxemia even when bacterial translocation across the gut epithelium is modest. Concurrently, the gut microbiome undergoes a pathological shift termed “dysbiosis”, which is characterized by a reduction in beneficial autochthonous taxa (*Lachnospiraceae*, *Ruminococcaceae*) and a massive expansion of potentially pathogenic “pathobionts,” predominantly *Enterobacteriaceae* and *Enterococcaceae* [4]. This “Gram-negative bloom” is clinically catastrophic. The translocation of these pathobionts and their endotoxins into the portal circulation triggers a persistent systemic inflammatory response. This chronic hyperstimulation eventually leads to cirrhosis-associated immune dysfunction (CAID), a state of immune exhaustion where the host becomes incapable of clearing infections, leading to fatal sepsis and organ failure [5].

While gut dysbiosis is well-documented, a critical and under-investigated dimension is the “Resistome”, which is the aggregate reservoir of antimicrobial resistance (AMR) genes (ARGs) harbored within the gut microbiota. India, often termed the “AMR capital of the world,” faces a unique challenge where the gut microbiome of patients is densely populated with multidrug-resistant (MDR) organisms due to high environmental transmission and antibiotic exposure [6]. Recent surveillance from Indian ICUs reveals alarmingly high resistance rates: 84% resistance to third-generation cephalosporins and over 47% resistance to carbapenems among *Enterobacteriaceae*. Unlike in the West, where VRE (Vancomycin-Resistant *Enterococci*) is a major concern, the Indian landscape is dominated by carbapenem-resistant *Klebsiella pneumoniae* and *Acinetobacter baumannii* carrying *blaNDM*-1 and *blaOXA* genes. Consequently, when bacterial translocation occurs in these patients, it often involves organisms that are resistant to standard empiric antibiotics (ceftriaxone or piperacillin-tazobactam), leading to rapid clinical deterioration and high mortality [7,8]. Despite the clear clinical urgency, there are profound gaps in our understanding of the gut–immune–resistome axis, specifically within the Indian population. Most existing studies on the cirrhotic microbiome in India have relied on 16S rRNA sequencing. While this technique identifies who is present (taxonomy), it cannot identify what functional genes they carry. Consequently, the actual burden of “silent” antimicrobial resistance genes (the resistome) remains unquantified.

We currently lack data on the density of specific resistance determinants in the gut of Indian patients with cirrhosis and how they correlate with phenotypic resistance in blood cultures. While we know that systemic inflammation drives immune exhaustion, there is no data linking this immuneparesis directly to the gut ARG burden. We hypothesize that a dense gut antimicrobial resistance gene burden serves as a surrogate marker for colonization by multidrug-resistant pathobionts that are enriched in virulence factors and endotoxin production capacity. Chronic subclinical translocation of these MDR organisms and their PAMPs (lipopolysaccharide from Gram-negatives, lipoteichoic acid from *Enterococci*) drives persistent TLR4/TLR2 activation on monocytes and macrophages, leading to endotoxin tolerance—a state of epigenetic reprogramming characterized by downregulation of HLA-DR expression and impaired antigen presentation. Possibly, the ARG burden does not itself cause immune paresis, but rather reflects the density of the MDR pathobiont population that is the proximate driver of chronic immune exhaustion through sustained endotoxin exposure. It remains unknown if a higher load of resistance genes or specific pathobionts (like *Klebsiella*) acts as a stronger driver of T-cell exhaustion than general dysbiosis. The current literature often studies these components in isolation—either the microbiome, the resistance patterns, or the immune response. There is a lack of “multi-omics” studies in India that integrate metagenomics (bugs and genes) with immunophenotyping (host response) to create a comprehensive idea about outcome associations. The current study aimed to bridge these critical lacunae. It is the first of its kind in India to utilize shotgun metagenomics to comprehensively map the bacterial microbiota and associated resistome in hospitalized cirrhosis patients and correlate it with clinical outcomes, immune function and systemic inflammation. Understanding this axis is essential for developing precision interventions that could help decolonize MDR pathogens and reverse immune paralysis in this high-risk cirrhosis population.

## 2. Materials and Methods

### 2.1. Patients

We conducted a retrospective analysis of prospectively collected samples and clinical data from a tertiary care, referral and teaching hospital in South India. Our institution maintains a prospective biobank protocol wherein all hospitalized patients with cirrhosis are approached within 24 h of admission for participation in ongoing hepatology research. Written informed consent is obtained at this time for: (a) collection and storage of biological samples (stool, blood) for future research studies, (b) access to clinical data, and (c) long-term follow-up for outcome assessment. Regarding sample collection timing, two distinct temporal anchors should be noted. First, with respect to hospital admission: all stool samples were strictly collected within 24 h of hospital admission. Second, with respect to antibiotic initiation: in the majority of patients, stool collection was performed prior to the initiation of in-hospital antibiotics. However, in emergent cases where immediate antimicrobial therapy was clinically mandated upon presentation (e.g., suspected spontaneous bacterial peritonitis or sepsis), stool samples were collected after antibiotic initiation but still within the 24 h admission window. Thus, while the admission-relative timing was uniform across all patients (within 24 h), the antibiotic-relative timing varied depending on clinical acuity, with some samples collected pre-antibiotic and others post-antibiotic initiation. Samples were collected using sterile containers and stored at −80 °C. For the current study, we retrospectively identified patients from this biobank who had complete inflammatory biomarker data and adequate stool sample volumes, then performed shotgun metagenomic sequencing on these stored samples. To ensure immunological homogeneity, patients with HIV, those on immunosuppressive regimens, and those with hepatic and extra-hepatic malignancies were excluded. All patients were on oral or enteral nutrition at the time of stool sample collection. Patients with hepatic encephalopathy received diets per institutional protocol; others received standard ward diets. No patients were on exclusive parenteral nutrition at the time of stool collection. Detailed dietary assessment including caloric intake, macronutrient distribution, and fiber intake was not systematically performed. Cytokine profiling was performed using a multiplex immunoassay (Randox Cytokine Array Platform, Randox Laboratories, Crumlin, Northern Ireland, UK) to quantify twelve analytes, including interleukins (IL-1alpha, IL-1beta, IL-2, IL-4, IL-6, IL-8, IL-10), IFN-gamma, TNF-alpha, EGF, VEGF, and MCP-1). Assay precision was verified with intra- and inter-assay coefficients of variation maintained below 10% and 15%, respectively. Immunophenotyping via flow cytometry (Beckman Coulter, Indianapolis, IN, USA) assessed surface expression (Mean Fluorescence Intensity) of neutrophil CD64, monocyte HLA-DR, and CD14; these values were subsequently used to calculate the Sepsis Index. The study cohort was stratified into 30 distinct clinical (based on presence or absence of complication and disease severity) and investigational (based on median cut-offs) groups. The study comparisons examined multiple dimensions of cirrhosis patients, beginning with etiology (alcohol-associated versus MASLD) and hepatic function markers including presence and severity of jaundice. Mortality was ascertained through hospital records, outpatient follow-up, and telephonic follow-up with patients or next-of-kin. However, independent verification of cause of death through death certificates or autopsy was not uniformly available. The study protocol was approved by the Institutional Ethics Committee of Rajagiri Hospital (Approval No. RAJH2025/112b, dated 12 November 2025) in accordance with the Declaration of Helsinki. As the study utilized de-identified data and stored biological samples, the requirement for individual informed consent for this specific analysis was waived by the Ethics Committee.

### 2.2. General Statistical Analysis

Statistical analyses were conducted using R (version 4.3.0; R Foundation for Statistical Computing, Vienna, Austria) and MedCalc Software (version 23.3.5; Ostend, Belgium). AI-assisted formatting tools, specifically Gemini-3 (Google AI, Mountainview, California, USA) and Nano-Banana Pro (Google DeepMind, London, UK), were used exclusively for the visual presentation and formatting of tables and graphical summaries from the statistical output generated by R and MedCalc. These tools were not involved in data analysis, statistical computation, or interpretation of results. Their use was limited to converting raw statistical outputs into publication-ready formatted tables and figure layouts. All AI-formatted outputs were manually reviewed and verified against the original software-generated results by two investigators (CAP and TTO) to ensure accuracy and fidelity. No AI-generated content was included without human validation. Comprehensive descriptive statistics were computed for each variable across comparison groups, including mean ± standard deviation (SD), standard error, and median accompanied by interquartile range (IQR; 25th–75th percentile, determined via linear interpolation when required). Results were reported as mean ± SD for parametric data and as median (IQR) for non-parametric data. Given the exploratory nature of this study—which aimed to generate hypotheses regarding the gut–immune–resistome axis rather than confirm pre-specified associations, formal multiple-comparison correction was not applied across the clinical group comparisons. Instead, a dual-threshold approach was employed, requiring both nominal statistical significance (*p* < 0.05) and a biologically meaningful effect size (log2 fold change ≥ 1) to reduce the likelihood of reporting spurious associations. This strategy was chosen to balance the risk of type I error against the risk of type II error (failing to detect biologically relevant signals) in a hypothesis-generating study of this scale.

### 2.3. Genomic Sequencing and General Bioinformatics

Stool DNA was extracted using the QIAamp PowerFecal Pro DNA Kit (Qiagen N.V., Hilden, Germany), utilizing bead-beating mechanical lysis and silica-column purification, followed by quantification via the Qubit dsDNA High Sensitivity Assay. Sequencing libraries were constructed using the Twist Enzymatic Fragmentation 2.0 kit (Twist Biosciences, South San Francisco, CA, USA) and sequenced on the Illumina NovaSeq X Plus (Illumina Inc., San Diego, CA, USA) platform (2 × 150 bp). Raw data underwent quality assessment using FastQC and MultiQC, adapter trimming with fastq-mcf (v1.04.803), and human host sequence removal via alignment to the human reference genome (GRCh38) using BWA-MEM. Quality-filtered, host-depleted reads were directly classified (without assembly) using DIAMOND BLASTX against the NCBI NR database. Taxonomic assignments were determined using the lowest common ancestor (LCA) algorithm implemented in MEGAN6. Read counts per taxon were used to calculate relative abundances. For functional gene prediction, non-host reads were de novo assembled using MEGAHIT (v3.11.1) to generate contigs, followed by Open Reading Frame (ORF) prediction using Prodigal (v2.6.3). For antimicrobial resistance gene quantification, adapter-trimmed paired-end reads were aligned directly (without assembly) to the Comprehensive Antibiotic Resistance Database (CARD) using Bowtie2 (v2.5.4) with very-sensitive-local parameters. Alignment files were processed using Samtools (v1.21) and mapped read counts were calculated using featureCounts. For both taxonomic and resistome data, raw read counts were normalized using DESeq2’s median-of-ratios method to account for library size variation across samples. Differential abundance testing between clinical groups was performed using Wald tests within DESeq2, with significance defined as *p* < 0.05 and log2 fold change ≥1. with visualizations including heatmaps, volcano plots, and error bar plots generated using ggplot2.

## 3. Results

### 3.1. Patients

The study cohort comprised 78 hospitalized patients (Supplementary Table S1) with cirrhosis, with a mean age of 58.74 ± 9.72 years and a marked male predominance (87.2%). The predominant etiology was metabolic dysfunction-associated steatotic liver disease (MASLD), accounting for 55.1% of cases, followed by alcohol-related cirrhosis in 38.5% of patients. The cohort demonstrated significant disease severity, with a mean MELD3 score of 25.14 ± 7.34 and median Child–Turcotte–Pugh score was 9 (IQR: 8–10) with distribution by class: Child–Pugh Class A 7.7% (n = 6), Class B 55.1% (n = 43), and Class C 37.2% (n = 29). At admission, ascites was present in 45 patients (57.7%), while 30 (38.5%) had hyponatremia (serum sodium < 130 mEq/L) and 25 (32.1%) presented with clinical jaundice. Active infection complicated the presentation in 21 patients (26.9%), with acute kidney injury (per AKIN criteria) documented in 14 (17.9%). Among patients with documented infections at admission, spontaneous bacterial peritonitis (SBP) was most common (38.1%, n = 8), followed by urinary tract infection (23.8%, n = 5), pneumonia (19.0%, n = 4), skin/soft tissue infection (9.5%, n = 2), and bacteremia without identified focus (9.5%, n = 2). Cultures (blood, ascitic fluid, urine, or wound swab, as clinically indicated) were positive in 14 of 21 infected patients (66.7%). Isolated organisms included *Escherichia coli* (n = 5), *Klebsiella pneumoniae* (n = 4), *Enterococcus* species (n = 3), and *Staphylococcus aureus* (n = 2). Among Gram-negative isolates, 66.7% (6/9) demonstrated extended-spectrum beta-lactamase (ESBL) production, and 22.2% (2/9) were carbapenem-resistant. Acute variceal bleeding was the presenting complaint in 13 (16.7%) cases, and hepatic encephalopathy (West-Haven grade ≥ 2) in 10 (12.8%). Acute-on-chronic liver failure was diagnosed at admission in 15 patients (19.2%). ICU admission was required in 30 patients (38.5%). Long-term mortality at 12–24 months follow-up was 43.6% (34/78 patients). Comprehensive inflammatory profiling revealed significant elevations across multiple cytokine families. IL-6 demonstrated the highest absolute levels (median 98.73 pg/mL, IQR: 47.22–190.79), with 52.6% of patients exceeding normal ranges. TNF-α was elevated in 48.7% of patients (median 18.13 pg/) mL, while IL-1β elevation occurred in 41.0%. The anti-inflammatory cytokine IL-10 was elevated (>9.1 pg/mL) in 29.5% of patients (n = 23) with median levels of 19.28 pg/mL. Among chemokines, MCP-1 showed marked elevation (median 284 pg/)mL in 55.1% of patients. Flow cytometry-based immune cell markers revealed elevated nCD64 expression in 36.2% and elevated Sepsis Index in 31.9% of patients.

### 3.2. Overall Bacterial Taxonomic Abundance and General Associations

Microbiome analysis across the clinical, inflammatory, and immune function cohorts revealed a consistent dysbiosis signature correlating with disease severity and systemic inflammation. Patients with ALD etiology and markers of higher disease severity (high MELD scores ≥ 25, Child–Pugh Class C, ACLF) and clinical complications such as ascites, hepatic encephalopathy, infection, gastrointestinal bleeding, and acute kidney injury exhibited a profound expansion of the classes Bacilli and Gammaproteobacteria. This specific taxonomic shift mirrored the profiles observed in patients with elevated inflammatory cytokines (IL-1alpha, IL-1beta, IL-2, IL-4, IL-6, IL-8, IL-10, IFN-gamma, TNF-alpha, MCP-1), high systemic inflammation indices, and markers of immune paralysis (low mHLA-DR and mCD14). At the family level, these severe and inflammatory states were dominated by *Enterobacteriaceae*, *Enterococcaceae*, and *Lactobacillaceae*, driven by the enrichment of potentially pathogenic species including *Klebsiella pneumoniae*, *Enterococcus faecium*, *Escherichia coli*, and *Ligilactobacillus salivarius*. Conversely, patients with lower disease severity markers (Child–Pugh Class B, MELD < 25), survivors at follow-up, and those not requiring ICU admission, and higher levels of regenerative growth factors (EGF and VEGF) retained a dominance of Bacteroidia and Clostridia, characterized by the preservation of beneficial families like *Lachnospiraceae*, *Ruminococcaceae*, and *Bacteroidaceae*, and key commensal species such as *Faecalibacterium prausnitzii* and various *Bacteroides* species (e.g., *B. thetaiotaomicron*, *B. uniformis*, *B. fragilis*) When analysed by etiology independently, MASLD patients showed relatively preserved commensal populations compared to ALD patients (Table 1, Supplementary Figure S1).

### 3.3. Specific Genus and Species Level Abundance Profiles

**Figure 1:**
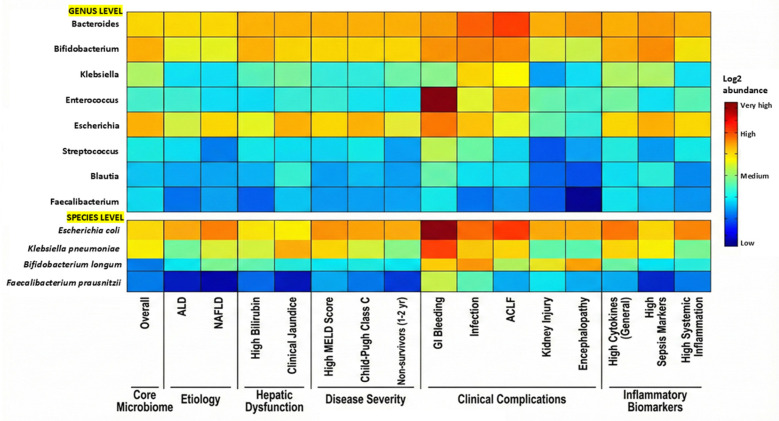
Heatmap depicting log2-transformed relative abundance of gut microbiota at the genus and species levels across clinical parameters in cirrhosis patients. The columns represent the core microbiome composition (overall), etiology, markers of hepatic dysfunction, disease severity indices, clinical complications and inflammatory biomarkers. The colour gradient ranges from blue (low abundance) to red (very high abundance), illustrating distinct microbial signatures associated with disease severity and complications.

Heatmap analysis revealed patterns consistent with a core dysbiosis microbiome signature across the cohort, characterized by the co-existence of the detectable commensal genera Bacteroides and Bifidobacterium alongside a highly abundant pathobiont cluster comprising *Klebsiella*, *Enterococcus*, and *Escherichia*. This pattern potentially represents a state of partial dysbiosis with commensal–pathobiont co-existence rather than complete commensal replacement, consistent with the spectrum of disease severity in our cohort (CTP Class B–C). This microbial landscape was dynamically modulated by clinical severity; patients with advanced hepatic decompensation, including high bilirubin, elevated MELD scores, Child–Pugh Class C status, and mortality, demonstrated a shift toward facultative anaerobes, where *Enterococcus* and *Klebsiella* abundances rivalled those of Bacteroides. Notably, gastrointestinal bleeding was associated with a specific, massive expansion of Enterococcus to co-dominant levels, while complications such as infection, acute-on-chronic liver failure, and acute kidney injury were similarly linked to high burdens of *Klebsiella pneumoniae*, *Escherichia coli*, and *Enterococcus faecium*. This enrichment of pathobionts paralleled high inflammatory states, with elevated cytokines, sepsis markers (CD64, mHLA-DR), and systemic inflammation indices consistently associated with the dominance of these organism-associated taxa over beneficial groups like *Faecalibacterium* and *Blautia* (Figure 1).

### 3.4. Analysis of Significant Bacterial Taxonomic Communities Between Groups

**Figure 2:**
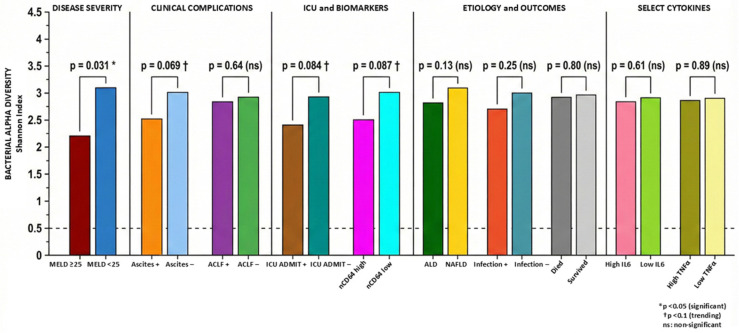
Bar graph illustrating bacterial alpha diversity, measured by the Shannon Index, across clinical subgroups in cirrhosis patients. Comparisons are organized into five categories: disease severity, clinical complications, acute-on-chronic liver failure, ICU and biomarkers, etiology and outcomes and select cytokines. Patients with MELD ≥ 25 demonstrated significantly lower alpha diversity compared to those with MELD < 25 (*p* = 0.031). Trending associations († *p* < 0.1) were observed for ascites (*p* = 0.069), ICU admission (*p* = 0.084), and high nCD64 expression (*p* = 0.087). No significant differences were found for ACLF, etiology, infection status, mortality, or cytokine levels. The dashed horizontal line indicates a Shannon Index threshold of 0.5. Statistical significance: * *p* < 0.05; † *p* < 0.1 (trending); ns: non-significant.

The log2 fold change analysis demonstrated that advanced hepatic dysfunction and severe clinical complications are primarily characterized by the significant depletion of diverse commensal taxa, specifically *Mycolicibacterium* and *Mycobacterium* species, *Nakamurella multipartita*, *Methylobacterium radiotolerans*, and *Blautia* species (*B. marasmi*, *B. hansenii*), a pattern observed consistently across groups with high bilirubin, acute kidney injury, ACLF, infection, and mortality. While poor outcomes (mortality) and ALD etiology were linked to the loss of beneficial microbes like *Prevotella copri*, this species was paradoxically enriched in hepatic encephalopathy alongside *Streptococcus thermophilus*. In terms of pathogenic enrichment, *Clostridium* sp. C5-48 emerged as a marker of severe decompensation (correlated with jaundice, Child–Pugh Class C and ascites), whereas *Sutterella* sp. AM11-39 was elevated in patients with ascites and high inflammatory cytokines (IL-6 and IL-8). Distinct immune profiles were further differentiated by specific taxa, including the enrichment of *Lactococcus lactis* in high MELD and TNF-alpha cohorts, and the association of *Akkermansia muciniphila* with elevated IFN-gamma levels (Figure 2).

### 3.5. Antimicrobial Resistance Gene Expression Between Groups

Heatmap analysis of the resistome characterized a pervasive background of tetracycline [*tet(M)*, *tet(O)*] and macrolide (*ErmX*) resistance across the cohort, particularly in patients with ALD; however, this profile underwent distinct compositional shifts correlating with clinical severity and inflammatory burden. Patients exhibiting markers of advanced hepatic decompensation, specifically high bilirubin, clinical jaundice, elevated MELD scores, Child–Pugh Class C, and ACLF, show a marked enrichment of the quinolone resistance gene *QnrB4* and the multidrug efflux pump *efmA*, signalling a transition toward a more resistant phenotype in critical illness. Notably, specific complications drove unique signatures: hepatic encephalopathy was associated with the highest intensity of gene expression and a distinct expansion of InuB, while gastrointestinal bleeding correlated with elevated levels of the beta-lactamase *OXA-833*. This escalation in resistance gene burden, particularly *tet(M)* and *efmA*, was universally mirrored in patients with active infection, high neutrophil CD64 expression, and elevated systemic inflammation indices, as well as across the full spectrum of pro-inflammatory cytokine profiles (including IL-1beta, IL-6, and TNF-alpha), confirming a link between systemic immune activation and the expansion of the gut resistome (Supplementary Figure S2).

Analysis of AMR gene abundance revealed distinct signatures correlating with disease severity, complications, and inflammatory status. Markers of advanced hepatic dysfunction, including high MELD scores (≥25), Child–Turcotte–Pugh Class C, ACLF, and significant complications such as hepatic encephalopathy, acute kidney injury, and gastrointestinal bleeding, were consistently associated with elevated abundances of *ErmX* and *tet(O)*. In contrast, phenotypes characterized by milder disease (Child Class B, low MELD), the absence of infection or organ failure, and preserved immune competence (high mHLA-DR, low Sepsis Index) exhibited significant enrichment of *tet(M)* and *efmA*. Etiologically, ALD-cirrhosis was specifically linked to *tet(O/W/32/O)* and *tet(O)*, whereas MASLD-cirrhosis favored *tet(M)* and *ErmX*. Furthermore, heightened inflammatory states (elevated IL-6, TNF-alpha, and CD64 expression) correlated with increased *ErmX*, *tet(O)*, and *aadA5*, while mortality at 1–2 years presented a unique profile characterized by a generalized upregulation of *tet(M)*, *ErmX*, *efmA*, and *tet(O)* in non-survivors, diverging from the specific gene-severity associations observed in surviving cohorts (Table 2).

**Figure 3:**
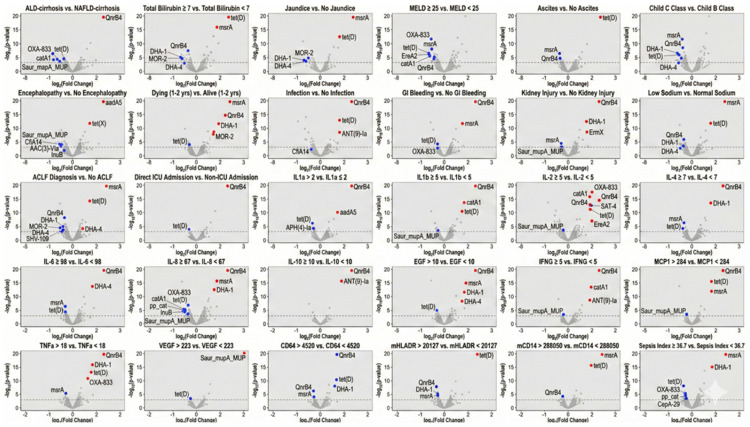
Volcano plots depicting differential abundance of antimicrobial resistance (AMR) genes across clinical, disease severity, and inflammatory biomarker subgroups in cirrhosis patients. Each panel displays log2 fold change (x-axis) versus −log10 *p*-value (y-axis), with significantly upregulated genes shown in red (positive fold change) and downregulated genes in blue (negative fold change); gray points represent non-significant findings. The horizontal dashed line indicates the significance threshold. Comparisons include: etiology, hepatic dysfunction markers, disease severity indices, clinical outcomes, ACLF, direct ICU admission, and inflammatory biomarkers stratified by median cutoffs. Prominent AMR genes differentially expressed include *QnrB4* (fluoroquinolone resistance), *tet(D)* (tetracycline resistance), msrA (macrolide resistance), *DHA-1* and *DHA-4* (*AmpC* β-lactamases), and *OXA-833* (oxacillinase β-lactamase).

On further analysis, *QnrB4* was significantly enriched in ALD-cirrhosis, whereas *tet(D)*, *OXA-833*, and *catA1* were more abundant in MASLD-cirrhosis. Poor clinical outcomes and complications, including 1–2-year mortality, infection, GI bleeding, kidney injury, and direct ICU admissions, were consistently characterized by the enrichment of *QnrB4*, often appearing alongside *msrA* and *DHA-1*. In contrast, *tet(D)* expression displayed a complex association profile; while linked to indicators of hepatic decompensation such as jaundice, ascites, and ACLF, it was simultaneously associated with survival at follow-up and non-ICU admission. A profound pro-inflammatory state (elevated IL-1beta, IL-6, IL-8, TNF–alpha, and IFN–gamma) correlated well with *QnrB4* upregulation, whereas *Saur_mupA_MUP* was frequently enriched in patients with lower cytokine profiles. Furthermore, markers of immune paralysis (lower CD64 and HLA-DR expression) were associated with the enrichment of *QnrB4*, *msrA*, and *DHA-1*, suggesting a distinct resistance landscape in immunoparetic patients compared to those with systemic inflammation (high SI), where *msrA* and *DHA*-1 were also upregulated (Figure 3).

## 4. Discussion

Our study represents a pioneering effort as the first study in India to utilize shotgun metagenomics to comprehensively map the bacterial microbiota and associated resistome in hospitalized sick cirrhosis patients. By integrating this high-resolution metagenomic data with detailed immunophenotyping (host response) and clinical outcomes, we delineated the specific microbial and genetic drivers of inflammation, immune exhaustion, and mortality in this high-risk population. In cirrhosis progression, the gut microbiome undergoes a pathological shift termed “dysbiosis.” This state is characterized by a reduction in beneficial autochthonous taxa, particularly from the families *Lachnospiraceae* and *Ruminococcaceae* (Class Clostridia), which are crucial for producing short-chain fatty acids like butyrate that maintain barrier integrity and immune homeostasis. In their place, there is a massive expansion of potentially pathogenic “pathobionts,” predominantly from the families *Enterobacteriaceae* and *Enterococcaceae*. This phenomenon, often described as the “Gram-negative bloom,” is clinically catastrophic [9].

A pivotal finding in our cohort is the predominance of MASLD (55.1%) as the leading etiology of hospitalized decompensated cirrhosis, surpassing ALD. This mirrors the rapid epidemiological transition reported in South Asia, where the adoption of Westernized diets and sedentary lifestyles has accelerated the prevalence of metabolic liver disease. The distinct microbiome signatures we observed in MASLD patients [enrichment of *tet(D)* and *catA1*] versus ALD patients (enrichment of *QnrB4*) suggest that etiology drives distinct resistome acquisition pathways, likely influencing response to prophylactic antibiotics.

The divergent resistome profiles between MASLD and ALD patients may reflect fundamentally different pathways of resistance gene acquisition. In ALD, the predominance of *QnrB4* (a plasmid-mediated quinolone resistance gene typically associated with *Klebsiella* and other *Enterobacteriaceae*) may be driven by the synergistic effects of alcohol on the gut barrier: ethanol directly increases intestinal permeability, promotes small intestinal bacterial overgrowth, and creates a bile acid milieu that selects for Gram-negative pathobionts intrinsically carrying quinolone resistance plasmids. Furthermore, ALD patients are frequently hospitalized with recurrent episodes requiring repeated antibiotic courses, creating sustained selective pressure favouring quinolone-resistant clones. In contrast, the enrichment of *tet(D)* and *catA1* (tetracycline and chloramphenicol resistance, respectively) in MASLD may reflect a different ecological niche: the metabolic environment of insulin resistance, hyperglycaemia, and altered bile acid composition characteristic of MASLD selects for a distinct microbial community. Tetracycline resistance genes are commonly found in agricultural and dietary microbiota, and the dietary patterns associated with metabolic syndrome (high-fat, processed foods) may introduce or select for organisms carrying these environmental resistance determinants. These etiology-specific resistome profiles have direct implications for prophylactic antibiotic selection, suggesting that norfloxacin prophylaxis may be particularly ineffective in ALD patients harboring *QnrB4*-enriched flora.

In our cohort of 78 hospitalized Indian patients with cirrhosis characterized by high disease severity and long-term mortality, the results revealed a profound gut microbiome dysbiosis marked by a significant expansion of Bacilli and Gammaproteobacteria, specifically *Enterobacteriaceae* and *Enterococcaceae*, in subjects with advanced hepatic dysfunction and complications. This pathological shift, driven by the enrichment of pathobionts such as *Klebsiella pneumoniae*, *Escherichia coli*, and *Enterococcus faecium*, mirrored profiles of systemic inflammation (elevated IL-6, TNF-alpha) and immune paralysis (low HLA-DR). Resistome profiling demonstrated that while a background of tetracycline and macrolide resistance was pervasive, critical illness and poor outcomes (including mortality, infection, and ACLF presentations) were distinctly characterized by the enrichment of the quinolone resistance gene *QnrB4* and multidrug efflux pumps like *efmA*. Notably, *QnrB4* expression plausibly correlated with a pro-inflammatory cytokine storm and immune paresis, whereas *tet(D)* displayed a complex duality, associated with both hepatic decompensation markers and patient survival.

The microbial landscape in Indian hospitals differs significantly from the West. While Western ICUs often struggle with Vancomycin-Resistant *Enterococci* (VRE) and *Clostridioides difficile*, the Indian landscape is dominated by Gram-negative bacteria, particularly *Klebsiella pneumoniae*, *Escherichia coli*, and *Acinetobacter baumannii*, which exhibit alarmingly high rates of resistance to third-generation cephalosporins (up to 84%) and carbapenems (over 47%). These organisms frequently carry potent resistance determinants such as *blaNDM-1* (New Delhi Metallo-beta-lactamase) and various *blaOXA* carbapenemase. Consequently, when bacterial translocation occurs in Indian patients with cirrhosis, it often involves organisms that are resistant to standard empiric antibiotics (e.g., ceftriaxone or piperacillin-tazobactam), leading to rapid treatment failure, clinical deterioration, and high mortality [10]. The 12–24-month mortality rate in our study was substantially high at 43.6%, with 38.5% of patients requiring direct ICU admission. This high mortality rate underscores the fact that the physiological reserve was depleted, and the window for therapeutic intervention is narrow. The distribution of complications, particularly the high rates of ascites and infection, points toward our cohort as one with severe portal hypertension and significant immune paresis.

Notably, elevated neutrophil CD64 expression (a validated marker of neutrophil activation in sepsis) was observed in some patients who did not have clinically or microbiologically confirmed infections (i.e., culture-negative). This apparent paradox likely reflects a state of ‘sterile’ systemic inflammation, wherein subclinical bacterial translocation from the gut (passage of viable bacteria or bacterial products such as LPS and bacterial DNA across the compromised intestinal barrier) triggers innate immune activation without progressing to overt bacteremia detectable by standard blood cultures. In cirrhosis, this phenomenon is well-described: portal hypertensive enteropathy and increased intestinal permeability allow continuous low-grade translocation of gut-derived PAMPs that activate neutrophils and monocytes via toll-like receptors, resulting in elevated nCD64, raised inflammatory cytokines, and clinical features mimicking sepsis in the absence of a positive culture. This ‘culture-negative inflammatory state’ is clinically significant because it may progress to overt sepsis and contributes to the hemodynamic instability characteristic of decompensated cirrhosis.

While high MELD scores (≥25) were associated with significantly reduced Shannon (alpha) diversity, several clinically important subgroups including ACLF showed no significant difference. These finding warrants explanation. First, dysbiosis in cirrhosis may manifest primarily as compositional shifts (changes in which taxa dominate) rather than overall diversity collapse. The Shannon Index captures both richness and evenness but may miss clinically relevant compositional changes when pathobiont expansion occurs alongside retained (but suppressed) commensals. Our data suggest that even in ACLF, patients retain detectable commensal populations while experiencing pathobiont expansion—a pattern of ‘partial dysbiosis’ rather than complete community collapse. Second, with only 15 ACLF patients (19.2% of cohort), statistical power to detect modest diversity differences was limited in our study. Third, ACLF encompasses heterogeneous precipitating events (infection, GI bleeding, hepatotoxins) that may have variable effects on microbiome diversity, potentially attenuating overall group differences. Thus, beta-diversity metrics examining community composition differences, rather than alpha-diversity, may be more informative for differentiating clinical phenotypes in this population.

Similarly, ICU admission, ascites, and elevated nCD64 showed only trending associations with reduced Shannon diversity that did not reach statistical significance. ICU admission is a heterogeneous endpoint reflecting various acute indications (GI bleeding, encephalopathy, respiratory failure) with potentially divergent microbiome effects. The near-significant trend for ascites supports a relationship with reduced diversity, though our binary classification (present/absent) rather than severity grading may have attenuated the signal. For nCD64, dichotomization of a continuous marker at the median may have reduced discriminatory power. Importantly, the absence of statistically significant alpha-diversity differences does not exclude important compositional differences. Indeed, differential abundance analyses revealed significant taxonomic and resistome differences in these groups, suggesting that community structure changes meaningfully even when overall diversity metrics are preserved.

The expansion of *Enterobacteriaceae* in our cohort with severe disease is consistent with the established literature documenting this phenomenon in cirrhosis. While the expansion of *Enterobacteriaceae* is a known hallmark of cirrhosis, our use of shotgun metagenomics allowed for the identification of species-level associations that provide new mechanistic insights. We identified *Sutterella* sp. AM11-39 as a possible marker for ascites and higher levels of inflammation. Sutterella species are generally considered commensals with mild pro-inflammatory potential. However, our findings align with animal models of cirrhosis where *Sutterella* translocation to ascitic fluid was linked to the onset of decompensation. This suggests *Sutterella* may possess specific bile-resistance traits allowing it to survive the altered bile acid milieu of cirrhotic patients, acting as a driver of peritoneal inflammation [11].

The association of *Clostridium* sp. C5-48 with severe decompensation and jaundice is a critical finding. This organism has been recently reclassified as *Enterocloster alcoholdehydrogenati*, a bacterium capable of producing high levels of carcinogenic acetaldehyde from ethanol. Its enrichment in our severe cohort could suggest a mechanism for “abstinential progression,” where the gut microbiota continues to generate hepatotoxic metabolites endogenously, perpetuating liver injury even in the absence of alcohol consumption [12].

Our study is among the first in India to map the gut resistome in decompensated cirrhosis, revealing a high burden of *QnrB4*, *OXA-833*, and *efmA*. The possible correlation between *QnrB4* (plasmid-mediated quinolone resistance) and mortality, ACLF, and immune paralysis (low HLA-DR) was striking. *QnrB4* is often co-located on multidrug-resistance plasmids with other virulence factors. Its presence likely signifies colonization by high-risk MDR clones (e.g., *Klebsiella* ST37 or ST11) that are endemic in India. The correlation with immune paralysis supports the hypothesis that chronic endotoxin exposure from these specific MDR pathobionts drives monocytes into a state of exhaustion (endotoxin tolerance), rendering the host defenseless against secondary infections [13,14].

The specific elevation of the carbapenemase gene *OXA*-*833* in patients with GI bleeding is a novel observation. *OXA*-*833* is an emerging resistance gene in South Asia, often found in *Proteus* and *Klebsiella* species [15]. This probably suggests that the nutrient-rich environment created by intraluminal blood (iron and protein) may exert a selective pressure favouring specific proteolytic, carbapenem-resistant organisms, complicating prophylactic antibiotic strategies in bleeders.

The multidrug efflux pump gene *efmA* was linked to Enterococcus faecium abundance. Interestingly, it was enriched in milder disease states (Child B) compared to genes like *ErmX* but was universally upregulated in non-survivors. The *efmA* is an intrinsic major facilitator superfamily (MFS) efflux pump in *E. faecium* that confers low-level resistance to fluoroquinolones and macrolides. The omnipresence of *efmA* in *E. faecium* strains confirms that the expanding *Enterococcus* populations in these patients are intrinsically equipped to survive antibiotic pressure [16]. The shift from *efmA* dominance to other genes like *ErmX* or *tet(O)* in specific complications (like HE) suggests a dynamic restructuring of the resistome under the selective pressure of different treatment regimens (e.g., rifaximin, which targets RNA polymerase but may select for other resistance mechanisms via plasmid co-selection).

The identification of *Enterococcus faecium* as a key pathobiont in our cohort warrants specific discussion, given that certain strains of this species are widely used as probiotics in commercially available formulations. It is essential to recognize that the species *E. faecium* encompasses genetically distinct lineages with fundamentally different clinical implications. Probiotic *E. faecium* strains typically belong to commensal-associated Clade B (e.g., ST296, ST67), which lack the virulence and resistance gene repertoire of hospital-adapted strains. In contrast, the *E. faecium* populations expanding in our decompensated cirrhosis patients were characterized by the enrichment of the *efmA* multidrug efflux pump, a hallmark of the hospital-adapted Clade A1 lineage (e.g., ST17, ST18, ST78) that carries intrinsic resistance to fluoroquinolones and macrolides. The co-existence of *efmA* enrichment with elevated TNF-alpha and markers of immune paralysis in our cohort suggests colonization by these pathogenic lineages rather than probiotic-type commensals. This distinction has critical clinical implications: the empirical use of *E. faecium*-containing probiotics in patients with advanced cirrhosis may potentially and inadvertently introduce organisms capable of acquiring vancomycin resistance (*vanA*/*vanB*) via horizontal gene transfer from co-resident pathobionts, potentially worsening the resistome burden. We therefore opine that clinicians exercise caution regarding probiotic formulations containing *E. faecium* in decompensated cirrhosis, pending species- and strain-level safety evaluation in this population.

Hepatic encephalopathy was associated with the highest intensity of AMR gene expression, specifically the lincosamide (e.g., clindamycin) resistance gene *lnuB*. *lnuB* encodes a lincosamide nucleotidyltransferase and is often found on mobile genetic elements (transposons and plasmids) in *Streptococcus* and *Enterococcus* species [17]. Overt HE treatment relies heavily on rifaximin (a rifamycin) and lactulose. While rifaximin resistance is a concern, the expansion of *lnuB* (which confers resistance to clindamycin/lincomycin) might be a collateral marker of the specific dysbiosis associated with HE (e.g., *Streptococcus salivarius* or *Enterococcus* overgrowth). It indicates that the gut flora in HE is not just ammoniagenic but also a dense reservoir of mobile resistance elements that could be transferred to pathogens. Additionally, HE linked to the highest AMR gene expression may also be due to several other factors: severe portal hypertension and portosystemic shunting in HE lead to reduced hepatic clearance of gut bacteria, while a distinct dysbiosis with urease-producing, ammonia-generating organisms could be common. Hepatic encephalopathy patients also have prolonged antibiotic exposure within hospitalizations, fostering resistant strains and gene transfer. Additionally, slower gut motility allows more time for plasmid and resistance gene exchange. Together, these factors possibly create a “resistance amplification niche,” accounting for the high AMR gene burden seen in HE.

Our study differentiated between the tetracycline resistance genes, *tet(M)* and *tet(O),* revealing distinct clinical associations. The *tet(M*) gene expression abundance was associated with milder disease (Child B), survival, and higher regenerative markers (EGF/VEGF). The *tet(M)* is a ribosomal protection protein often found in commensals like Firmicutes. Its presence in healthier patients likely reflects a preserved commensal flora (which naturally carries some resistance) rather than a pathogen bloom. Conversely, *tet(O)* was associated with severe disease (Child C, ACLF), ALD, and mortality. The *tet(O)* gene is frequently associated with pathogens like *Campylobacter* and *Streptococcus* [10]. The switch from *tet(M)* to *tet(O)* may represent a “resistome shift” from commensal-driven resistance to pathogen-driven resistance as cirrhosis progresses.

Our study provided compelling evidence for a direct mechanistic link between the gut resistome and host immune function. High AMR genes burden was not a silent passenger but correlated satisfactorily with systemic inflammation (high IL-6, TNF-alpha) and immune paralysis (low HLA-DR, high Sepsis Index). Our work also highlighted that not all dysbiosis was created equal and that different pathobionts drive different states within the systemic inflammation. For example, we observed that *Ligilactobacillus salivarius* abundance was significantly linked to IFN-gamma and IL-6, suggesting a Th1/Th17-type inflammatory response. This aligns with the potential for *L. salivarius* to act as an opportunistic pathogen driving cellular immunity. Similarly, *Enterococcus faecium* was ubiquitous in high TNF-alpha states. *E. faecium* lipoteichoic acid is a potent inducer of TNF-alpha via TLR2, contributing to the hemodynamic instability (vasodilation) seen in decompensated patients [18,19].

While this exploratory study requires prospective validation, several findings have potential clinical implications. First, the high burden of quinolone resistance genes (*QnrB4*) documented in our cohort suggests that norfloxacin, the current standard for spontaneous bacterial peritonitis prophylaxis, may be deleterious in patients with advanced decompensation. Second, the association of *QnrB4* with mortality and immune paralysis suggests potential utility as a prognostic biomarker; pending validation, stool-based resistome screening could identify patients requiring early ICU triage. Third, the association of *Ligilactobacillus salivarius* with pro-inflammatory cytokines (IL-6, IFN-gamma) challenges empirical use of generic *Lactobacillus*-containing probiotics in decompensated cirrhosis, warranting species-specific safety evaluation. Fourth, the enrichment of *OXA*-833 (carbapenemase) in patients with gastrointestinal bleeding suggests that standard ceftriaxone prophylaxis may be insufficient in this subgroup. Finally, the identification of specific pathobionts (*Clostridium* sp. C5-48, *Enterococcus faecium*) provides targets for precision microbiome interventions including bacteriophage therapy or targeted faecal microbiota transplantation.

From a translational perspective, the feasibility and cost of resistome-based screening merit discussion. Current shotgun metagenomic sequencing, as employed in our study, costs approximately USD 150–300 per sample (including DNA extraction, library preparation, and sequencing on Illumina platforms), with turnaround times of 5–7 days, which limits its utility for acute clinical decision-making. However, rapid technological advances are narrowing this gap. Targeted amplicon-based panels for specific resistance genes (such as *QnrB4*, *blaOXA*, and *blaNDM*) using real-time PCR can provide results within 4–6 h at a fraction of the cost (USD 30–50 per panel). In resource-limited settings like India, a tiered approach may be most practical: targeted PCR panels for high-risk resistance genes (*QnrB4*, *OXA-833*) as a rapid bedside screen, with comprehensive shotgun metagenomics reserved for research cohorts or patients with refractory infections. The cost-effectiveness of such screening relative to the economic burden of treating MDR infections in ICU settings (estimated at USD 5000–15,000 per episode in Indian tertiary centers) warrants formal health economic evaluation.

This study’s primary strength lies in its integration of shotgun metagenomics with deep immunophenotyping in an Indian cohort, allowing us to link specific resistome elements like *QnrB4* to immune states such as HLA-DR expression and revealing region-specific patterns distinct from Western cohorts. The detailed clinical stratification further enabled the identification of niche-specific associations, such as the unique resistome signature of GI bleeding versus hepatic encephalopathy. However, limitations include a modest sample size (n = 78) that restricts multivariate adjustments, a cross-sectional design that precludes causal inference regarding the resistome role in decompensation, and the analysis of luminal rather than mucosal microbiota. Dietary factors are important modulators of gut microbiome composition. While all patients were on oral/enteral nutrition at sample collection, detailed nutritional assessment (caloric intake, macronutrient distribution, fiber content) was not performed. Future studies should incorporate dietary questionnaires or food frequency data to assess diet-microbiome interactions in this population. Additionally, while current antibiotic use was recorded, the retrospective quantification of lifetime antibiotic exposure remains a challenge. It is imperative to note that the etiological distribution in our cohort (MASLD 55.1%, ALD 38.5%, with only 3.8% viral hepatitis) reflects the contemporary epidemiological landscape of cirrhosis at tertiary care centers in South India, where successful antiviral treatment programs have reduced viral hepatitis as a cause of decompensated cirrhosis. However, this limits generalizability to populations where viral hepatitis predominates. Some findings, such as Enterobacteriaceae expansion and resistome burden, likely relate to portal hypertension and gut barrier dysfunction common across etiologies, while others (e.g., *Clostridium* sp. C5-48 associations with ALD) may be etiology-specific. Validation in viral hepatitis cohorts is warranted before broader generalization.

Additionally, several potential confounders of the resistome were incompletely captured. While current antibiotic use (within 7 days of admission) and history of prior SBP were documented, we lack systematic data on: hospitalizations within the preceding 90 days, prior drug-resistant colonization/infection status, cumulative duration of antibiotic prophylaxis, and socioeconomic status. The omission of these factors is a limitation, as they may act as hidden drivers of the resistome. For instance, patients with frequent prior hospitalizations (unmeasured) may have a higher baseline load of multidrug-resistant organisms due to nosocomial exposure, potentially inflating the association between resistance genes and disease severity. Similarly, socioeconomic factors could influence environmental exposure to resistance determinants. Furthermore, due to the modest sample size relative to the high dimensionality of metagenomic data, we did not perform multivariate regression adjusting for age or these unmeasured confounders to avoid overfitting. Consequently, the associations reported herein should be interpreted as descriptive of the clinical phenotype rather than causally isolated.

An important consideration in interpreting our resistome findings is that the presence of an antimicrobial resistance gene in metagenomic data does not automatically confer phenotypic resistance. Our study detected DNA (gene presence) rather than RNA (gene expression), and several factors determine whether detected genes result in functional resistance: (1) Gene expression—many ARGs may be present but transcriptionally silent or expressed below clinically relevant levels; (2) Genetic context—ARGs require appropriate promoters, regulatory elements, and in some cases accessory genes for functional expression; truncated genes or those lacking proper regulatory contexts may not confer resistance; (3) Host organism—an ARG in a non-pathogenic commensal may have different clinical implications than the same gene in a pathogen, though horizontal gene transfer potential remains a concern; (4) Gene variants—some variants of resistance genes may have reduced or absent functional activity. However, the genes we identified (*QnrB4*, *OXA-833*, *efmA*, tetracycline resistance genes) are well-characterized resistance determinants with established functional roles documented in CARD and peer-reviewed literature. Furthermore, the clinical correlations observed—*QnrB4* with mortality in patients likely receiving prophylactic quinolones, *OXA-833* with GI bleeding where cephalosporin prophylaxis is standard—provide circumstantial support for functional relevance. Nevertheless, phenotypic susceptibility testing of cultured clinical isolates would provide definitive confirmation, representing an inherent limitation of purely metagenomic approaches. Therefore, the resistance genes identified in this study (*QnrB4*, *OXA-833*, *efmA*) should be interpreted primarily as markers of colonization by resistant flora rather than as direct evidence of phenotypic resistance in bloodstream infections. Their clinical correlations, while biologically plausible, require prospective validation through paired metagenomic and phenotypic susceptibility testing of cultured isolates.

Furthermore, given that differential abundance testing was performed across 30 clinical comparisons without formal multiple-comparison correction, the risk of false-positive associations is non-trivial. Although a dual threshold of nominal *p* < 0.05 combined with log2 fold change ≥ 1 was employed to mitigate this risk, some of the reported gene-level associations may represent chance findings. Accordingly, readers should interpret individual associations—particularly those involving genes with borderline significance or smaller effect sizes—with appropriate caution. Prospective validation studies with pre-specified hypotheses, adequate sample sizes, and formal FDR correction are warranted before any of these associations are considered for clinical application.

These findings pave the way for resistome-based risk stratification, where rapid screening for genes like *QnrB4* could guide early ICU transfer or antibiotic escalation. The identification of specific pathobionts such as *Clostridium* sp. C5-48 and *Enterococcus faecium* highlights the potential for precision microbiome editing via bacteriophage therapy to reduce toxin and AMR burdens. Moreover, the high burden of quinolone resistance calls for revised prophylaxis guidelines, potentially replacing norfloxacin with non-antibiotic alternatives like *Lachnospiraceae*-enriched Faecal Microbiota Transplantation (FMT) or dietary interventions targeting metabolic substrates to restore colonization resistance.

## 5. Conclusions

Our work delineates the pertinent deleterious changes in the gut–liver axis in Indian patients with decompensated cirrhosis, revealing a pathogenic ecosystem characterized by *Klebsiella*, *Enterococcus*, and novel markers such as *Clostridium* sp. C5-48 and *Sutterella*. Crucially, the association of *Ligilactobacillus salivarius* with cytokine dysregulation cautions against the empirical use of generic probiotics. Furthermore, the identification of specific resistance genes (*QnrB4*, *OXA-833*) as predictors of mortality and gastrointestinal bleeding, respectively, establishes a direct link between the resistome and host immune paralysis. These findings underscore the necessity of region-specific, precision microbiome prospective studies on interventions such as phage therapy and faecal microbiota transplantation to mitigate the dual burden of liver failure and antimicrobial resistance.

## Figures and Tables

**Table 1:** Summary representing “core drivers” of bacterial taxa associations and differences across clinical, immune, and inflammatory spectra.

Taxa Level	Associated with Severe Disease Phenotype (High MELD, ACLF, Death) and ALD Etiology	Associated with Milder Disease Phenotype (Low MELD, No ACLF, Survival) and MASLD Etiology
Class	Bacilli and Gammaproteobacteria: Consistently expanded in decompensated cirrhosis and ACLF.	Bacteroidia and Clostridia: Dominant in stable patients and survivors; depleted in severe disease.
Family	*Enterobacteriaceae* The hallmark of dysbiosis in this cohort. *Enterococcaceae* linked to infection and mortality.	*Ruminococcaceae* and *Lachnospiraceae*: Short-chain fatty acid producers associated with gut barrier integrity.
Genus	*Klebsiella*, *Enterococcus*, *Lactobacillus*: Genera typically associated with pathogen blooms.	*Faecalibacterium*, *Bacteroides*: Genera associated with healthy microbiome function.
Species	*Klebsiella pneumoniae*, *Enterococcus faecium*, *Escherichia coli*: Core drivers of mortality and infection.	*Faecalibacterium prausnitzii*, *Bacteroides thetaiotaomicron*, *Bacteroides fragilis*: Associated with survival and lower disease scores.
Taxa Level	Drivers of Immune Dysfunction and Inflammation (High nCD64, High SI, Low HLA-DR/CD14).	Drivers of Immune Competence (Low nCD64, Low SI, High HLA-DR/CD14).
Class	Bacilli: Correlated with high Sepsis Index (SI).	Bacteroidia: Associated with preserved monocyte function (High mHLA-DR).
Family	*Enterococcaceae*: Expanded in patients with immune paralysis (low mHLA-DR).	*Bacteroidaceae*: Dominant in patients with lower markers of neutrophil activation.
Species	*Enterococcus faecium*, *Escherichia coli*, *Klebsiella pneumoniae*: The “blooming” species driving high SI and immune failure.	*Bacteroides uniformis*, *Bacteroides ovatus*: Associated with lower systemic inflammation.
Taxa Level	Drivers of Cytokine Storm (High IL-1beta, IL-6, IL-8, TNF-α).	Drivers of Homeostasis/Regeneration (High EGF, High VEGF, Low Cytokines).
Phylum	Proteobacteria and Firmicutes (Bacilli): Expansion correlates with peak inflammatory markers.	Bacteroidetes: Preservation of this phylum correlates with regenerative markers like EGF.
Family	*Lactobacillaceae*: Specifically elevated in high IFN-γ and IL-6 states. *Enterobacteriaceae*: Universal driver across almost all high interleukin groups.	*Lachnospiraceae*: Consistently depleted in high cytokine states; preserved in high EGF/VEGF groups.
Species	*Ligilactobacillus salivarius* and *L. fermentum*: Unique driver noted in high IL-6 and IFN-γ groups. *Enterococcus faecium*: Ubiquitous in high TNF-α and IL-1beta groups.	*Faecalibacterium prausnitzii*, *Bacteroides vulgatus* and *B. ovatus*: Their abundance is inversely proportional to inflammatory cytokine levels.

Footnote: For immunophenotyping stratification, ‘high’ and ‘low’ groups were defined by the cohort median values. Patients above the median were classified as ‘high’ and those at or below the median as ‘low’ for each marker (Supplementary Table S1).

**Table 2:** Key antimicrobial resistance genes expression abundance and clinical associations.

Gene	Class of Resistance	Associated Bacteria	Clinical Associations
*QnrB4*	Quinolone (Low-level)	*Klebsiella*, *Enterobacteriaceae*	ALD, mortality, ICU admission, ACLF, immune paralysis
*efmA*	Macrolide/Fluoroquinolone	*Enterococcus faecium*	Non-survivors, Child Class B, high Sepsis Index
*OXA-833*	Beta-lactam (Carbapenemase)	*Proteus*, *Enterobacteriaceae*	Gastrointestinal bleeding, MASLD
*lnuB*	Lincosamide	*Streptococcus*, *Enterococcus*	Hepatic encephalopathy, hyponatremia
*tet(M)*	Tetracycline (Ribosomal)	*Firmicutes* (diverse)	Milder disease, survival, high EGF/VEGF
*tet(O)*	Tetracycline (Ribosomal)	*Campylobacter*, *Firmicutes*	Severe disease (Child C, ACLF), ALD

## Data Availability

The datasets generated during this study are not publicly available due to ethical restrictions and patient privacy considerations, as the institutional ethics approval did not permit open data deposition. Anonymized data supporting the findings are available from the corresponding author upon reasonable request, subject to institutional data sharing agreements.

## Supplementary Materials

The following supporting information can be downloaded at: https://www.mdpi.com/article/10.3390/pathogens15030241/s1, Figure S1: Summary of the most prominent bacterial taxa associated with clinical, immune function and inflammatory events in hospitalized cirrhosis patients, and Figure S2: Heatmap displaying the relative association intensity between antimicrobial resistance (AMR) genes and clinical, immune function, and inflammatory parameters in cirrhosis patients; Table S1: Baseline Demographic, Clinical, Laboratory, and Inflammatory Characteristics of the Study Cohort.

## Abbreviations

The following abbreviations are used in this manuscript:

ACLF	Acute-on-Chronic Liver Failure
ALD	Alcohol-related Liver Disease
AMR	Antimicrobial Resistance
ARG	Antimicrobial Resistance Genes
CAID	Cirrhosis-Associated Immune Dysfunction
CARD	Comprehensive Antibiotic Resistance Database
EGF	Epidermal Growth Factor
ESBL	Extended-Spectrum Beta-Lactamase
FMT	Faecal Microbiota Transplantation
HE	Hepatic Encephalopathy
HLA-DR	Human Leukocyte Antigen-DR
IFN	Interferon
IL	Interleukin
MASLD	Metabolic Dysfunction-Associated Steatotic Liver Disease
MCP-1	Monocyte Chemoattractant Protein-1
MDR	Multidrug-Resistant
MELD	Model for End-stage Liver Disease
TNF-alpha	Tumor Necrosis Factor-alpha
VEGF	Vascular Endothelial Growth Factor
