# Supplementary material for: Novel Insights on Clinical Outcomes Using Integrated Shotgun Metagenomic Profiling of the Gut Microbiome, Resistome, and Host Immune-Inflammatory Response in Hospitalized Patients with Decompensated Cirrhosis
Source: Pathogens. 2026 Feb 24;15(3):241. doi: 10.3390/pathogens15030241 (PMC13029280; doi:10.3390/pathogens15030241)

Supplementary Table S1: Baseline Demographic, Clinical, Laboratory, and Inflammatory Characteristics of the Study Cohort

Parameter	Number (N = 78)
Demographics	
Age (years), mean ± SD	58.74 ± 9.72
Male sex, n (%)	68 (87.2)
Female sex, n (%)	10 (12.8)
Etiology of Cirrhosis	
MASLD (NASH), n (%)	43 (55.1)
Alcohol-related liver disease, n (%)	30 (38.5)
Hepatitis B virus, n (%)	3 (3.8)
Herb-induced liver injury (HILI), n (%)	1 (1.3)
Autoimmune hepatitis, n (%)	1 (1.3)
Disease Severity Indices	
MELD-3.0 score, mean ± SD	25.14 ± 7.34
Child-Turcotte-Pugh score, median (IQR)	9 (8–10)
Child-Pugh Class, n (%)	
Class A (≤6)	6 (7.7)
Class B (7–9)	43 (55.1)
Class C (≥10)	29 (37.2)
Clinical Complications at Admission	
Ascites, n (%)	45 (57.7)
Hyponatremia (Na <130 mEq/L), n (%)	30 (38.5)
Clinical jaundice, n (%)	25 (32.1)
Active infection at admission, n (%)	21 (26.9)
Acute-on-chronic liver failure, n (%)	15 (19.2)
Acute kidney injury (AKIN criteria), n (%)	14 (17.9)
Acute variceal bleeding, n (%)	13 (16.7)
Hepatic encephalopathy (West-Haven ≥2), n (%)	10 (12.8)
Hepatocellular carcinoma, n (%)	12 (15.4)
Active alcohol use disorder, n (%)	16 (20.5)
Septic shock, n (%)	2 (2.6)
Mechanical ventilation, n (%)	1 (1.3)

Infection Details (of 21 infected patients)	
Spontaneous bacterial peritonitis, n (%)	8 (38.1)
Urinary tract infection, n (%)	5 (23.8)
Pneumonia, n (%)	4 (19.0)
Skin/soft tissue infection, n (%)	2 (9.5)
Bacteraemia (no identified focus), n (%)	2 (9.5)
Culture-positive infections, n/N (%)	14/21 (66.7)
ESBL (Gram-negative isolates), n/N (%)	6/9 (66.7)
Carbapenem-resistant (Gram-negative), n/N (%)	2/9 (22.2)
Outcomes	
ICU admission, n (%)	30 (38.5)
In-hospital mortality, n (%)	2 (2.6)
Mortality at 12–24 months follow-up, n (%)	34 (43.6)
Laboratory Parameters at Admission	
Haemoglobin (g/dL), mean ± SD	10.50 ± 2.10
Total leucocyte count ($\times 10^3/\mu L$), median (IQR)	6.35 (4.93–8.40)
Platelet count ($\times 10^3/\mu L$), median (IQR)	120 (90–140)
Total bilirubin (mg/dL), median (IQR)	4.80 (2.10–7.83)
Serum albumin (g/dL), mean ± SD	2.74 ± 0.59
Serum sodium (mEq/L), mean ± SD	131.12 ± 6.70
Serum creatinine (mg/dL), median (IQR)	1.20 (0.90–1.58)
INR, mean ± SD	1.91 ± 0.64
CRP (mg/L), median (IQR)	21.10 (10.45–40.25)
Procalcitonin (ng/mL), median (IQR)	0.40 (0.18–0.95)
Inflammatory Cytokine Profile (pg/mL)	
IL-6, median (IQR)	98.73 (47.22–190.79)
IL-8, median (IQR)	67.06 (30.64–200.16)
TNF-α, median (IQR)	18.12 (4.40–28.26)
MCP-1, median (IQR)	284.00 (200.93–396.03)
IL-4, median (IQR)	6.64 (6.60–11.11)
IL-10, median (IQR)	1.80 (1.80–6.51)
EGF, median (IQR)	10.47 (2.90–27.52)

VEGF, median (IQR)	167.26 (40.67–370.26)
IL-1α, median (IQR)	0.80 (0.80–1.66)
IL-1β, median (IQR)	1.60 (1.60–3.83)
IL-2, median (IQR)	4.80 (4.80–4.80)
IFN-γ, median (IQR)	4.40 (4.40–4.40)
Immunophenotyping (Flow Cytometry)	
nCD64, MFI, median (IQR)	5,116 (2,440–10,971)
mHLA-DR, MFI, median (IQR)	21,212 (14,987–29,355)
mCD14, MFI, median (IQR)	320,558 (236,157–393,435)
Sepsis Index, median (IQR)	28.70 (11.90–65.40)

Abbreviations: MASLD, metabolic dysfunction-associated steatotic liver disease; NASH, non-alcoholic steatohepatitis; ALD, alcohol-related liver disease; MELD, Model for End-stage Liver Disease; IQR, interquartile range; CTP, Child-Turcotte-Pugh; ACLF, acute-on-chronic liver failure; AKIN, Acute Kidney Injury Network; ESBL, extended-spectrum beta-lactamase; ICU, intensive care unit; MFI, mean fluorescence intensity; IL, interleukin; TNF, tumour necrosis factor; MCP-1, monocyte chemoattractant protein-1; EGF, epidermal growth factor; VEGF, vascular endothelial growth factor; IFN, interferon; nCD64, neutrophil CD64; mHLA-DR, monocyte HLA-DR; SI, Sepsis Index.

Note: Continuous variables reported as mean ± SD (normally distributed) or median (IQR) (skewed). Categorical variables as n (%).

Supplementary Figure S1: Summary of the most prominent bacterial taxa associated with clinical, immune function and inflammatory events in hospitalized cirrhosis patients.

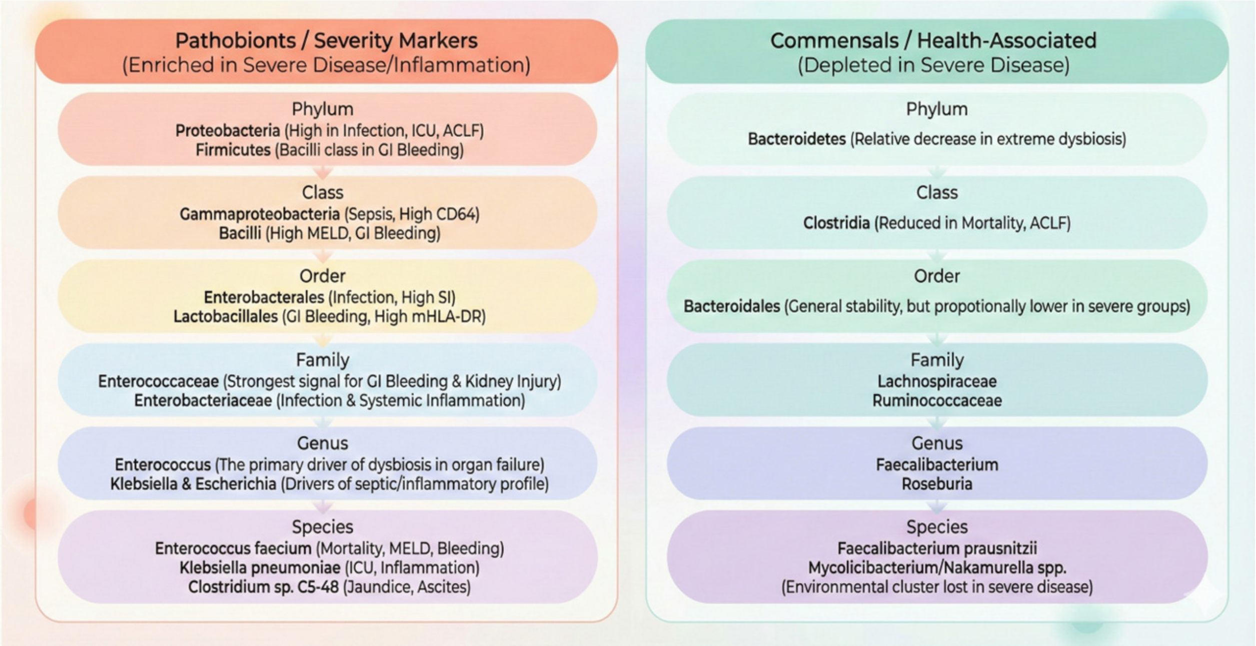

Supplementary Figure S2: Heatmap displaying the relative association intensity between antimicrobial resistance (AMR) genes and clinical, immune function, and inflammatory parameters in cirrhosis patients. The y-axis represents AMR genes including tetracycline resistance genes (tet(M), tet(O), tet(D)), macrolide resistance genes (ErmX, msrA), efflux pump genes (efmA), lincosamide resistance gene (lnuB), fluoroquinolone resistance gene (QnrB4), β-lactamase gene (OXA-833), streptothricin acetyltransferase gene (SAT-4), and *Klebsiella pneumoniae* outer membrane porin (Kpne_OmpK37). The x-axis depicts clinical variables: Etiology (ALD [Alcohol-related Liver Disease]), Bilirubin (High), Jaundice, MELD [Model for End-stage Liver Disease] (High), Ascites, Child-Pugh C, Encephalopathy, Mortality (Died), Infection, GI [Gastrointestinal] Bleeding, AKI [Acute Kidney Injury], Hyponatremia, ACLF [Acute-on-Chronic Liver Failure], ICU [Intensive Care Unit] Admission; cytokines and inflammatory markers: IL [Interleukin]-1a, IL-1b, IL-2, IL-4, IL-6, IL-8, IL-10, EGF [Epidermal Growth Factor], IFN-γ [Interferon-gamma], MCP1 [Monocyte Chemoattractant Protein-1], TNF-

α [Tumor Necrosis Factor-alpha], VEGF [Vascular Endothelial Growth Factor]; and immune cell markers: CD64, mHLA-DR [monocyte Human Leukocyte Antigen-DR], mCD14 [monocyte CD14], and SI [Systemic Inflammation] (High). The color gradient from light pink to dark purple indicates increasing relative association intensity (scale 0–5). Notable associations include tet(M), ErmX, and efmA genes showing broad associations across multiple inflammatory cytokines and clinical complications.

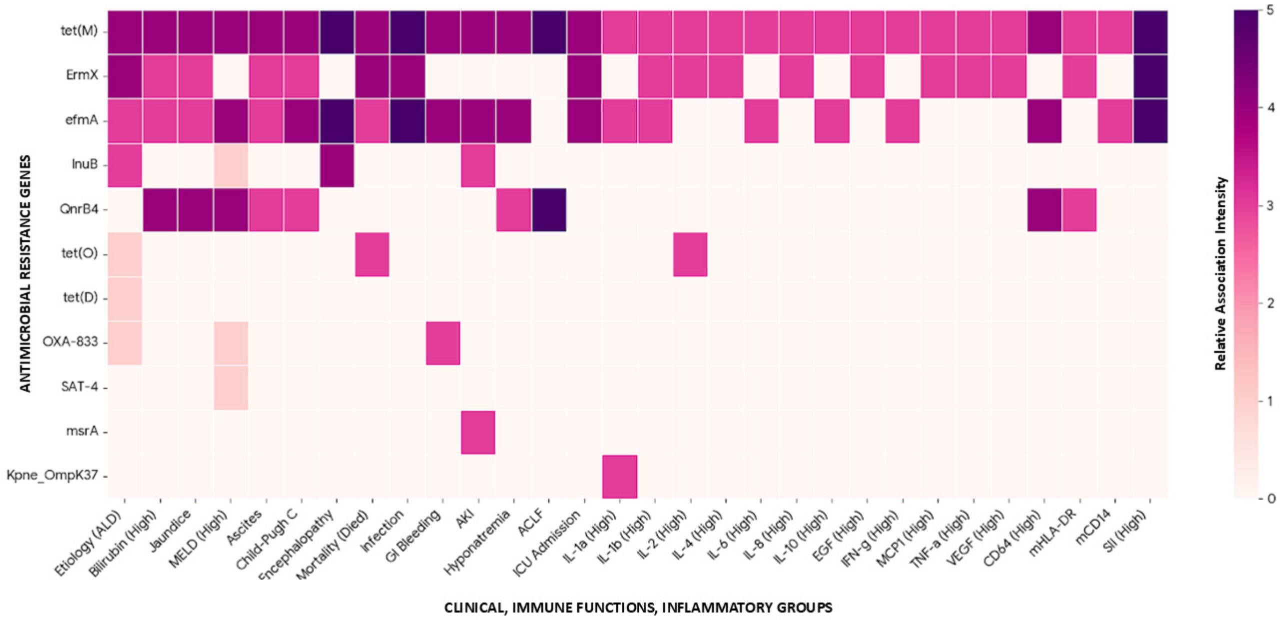

Supplement: Supplementary file 1 [file pathogens-15-00241-s001.zip › pathogens-4127156-supplementary.pdf]
